# Optimization of gamma‐aminobutyric acid production by *Lactobacillus brevis* PML1 in dairy sludge‐based culture medium through response surface methodology

**DOI:** 10.1002/fsn3.2304

**Published:** 2021-05-02

**Authors:** Fereshteh Falah, Alireza Vasiee, Behrooz Alizadeh Behbahani, Farideh Tabatabaee Yazdi, Seyed Ali Mortazavi

**Affiliations:** ^1^ Department of Food Science and Technology Faculty of Agriculture Ferdowsi University of Mashhad Mashhad Iran; ^2^ Department of Food Science and Technology Faculty of Animal Science and Food Technology Agricultural Sciences and Natural Resources University of Khuzestan Mollasani Iran

**Keywords:** dairy sludge, food waste, GABA, HPLC, *Lactobacillus brevis*

## Abstract

Gamma‐aminobutyric acid (GABA) is a pharmaceutical, bioactive amino acid that can produce by some species of Lactic Acid Bacteria (LAB). For the first time, we evaluated the production of GABA by *Lactobacillus brevis* PML1 in the medium that contain the contaminant food bio‐product like dairy sludge and soybean meal. GABA production was analyzed by chromatography (TLC, HPLC) and the features of fermented extract which contains this amino acid were evaluated. The results of Response Surface Methodology (RSM) of Central Composite Design (CCD) at *p* < .05 showed 300 ppm of GABA production in optimal treatment including 14.77% dairy sludge powder, 6.27% soybean meal, and 0.49% ammonium sulfate (32°C for 120 hr fermentation). The results of fermented extract also showed the acceptable antimicrobial, antioxidant, and toxicity (against cancer cell) properties. Also, *L. brevis* PML1has not shown any hemolytic or DNase activity which confirm its safety aspects. According to the results, this new culture can be used as a cheap substrate to biological production of GABA, by *L. brevis* PML1 in various food and pharmaceutical formulations.

## Graphical abstract

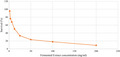

## 1 INTRODUCTION

Gamma‐aminobutyric acid (GABA) is a bioactive molecule with various physiological roles in the body. GABA has a inhibitory effect on the transmission of sympathetic nerve messages and, therefore, reduces neurological diseases. It also increases the synthesis of DNA and proteins in the brain and cell viability and by regulating blood pressure and increasing the body’s energy level, relieves fatigue, stress and anxiety. Stimulating the anterior pituitary gland, improving visual function, memory enhancement, reducing growth of some tumors, and inhibition of diabetes due to reduced destruction of beta‐pancreatic cells are other properties of this amino acid (Taylor & Tso, 2015).

Gamma‐aminobutyric acid is the end product of the decarboxylation of glutamic acid within lactic acid bacteria by Glutamic Acid Decarboxylase (GAD). This enzyme is produced by many microorganisms, including bacteria, mold, and yeasts. The catabolism pathway of glutamate is initiated by some enzymes like aminotransferase, dehydrogenase, or decarboxylase in the LAB. The activity of the first two enzymes results in the formation of alpha‐ketoglutarate from glutamic acid, while GABA production is formed by decarboxylase activity. Carbohydrates are converted to alpha‐ketoglutarate and ammonia by being placed in the Krebs cycle and then produced in the cytoplasm of the L‐glutamate cell and then converted to diglutamate and eventually to polyglutamic acid. During decarboxylation of glutamic acid, gamma‐aminobutyric acid is produced. GABA production begins in the bacterial growth phase and increases near the stationary phase due to increased GAD activity. It is an intracellular enzyme that is produced in response to acidic conditions (Atanda et al., 2007). *Lactobacillus brevis* is a type of LAB that is considered a safe bacterium and can be used in various fermentation cultures and production of beneficial products (Cai et al., 2014).

As the population grew and more energy resources were needed, various technologies were invented and applied to solve the problem as lack of resources. One of these solutions is the use of wastes from different industries in the production of useful and usable products. Many of these sources contain various compounds, including carbohydrates, proteins, vitamins (Sen et al., 2016).

Dairy waste as a strong wastewater with high oxygen concentration is a biological and chemical requirement that contains a lot of organic matter. This type of waste causes serious problems in the municipal wastewater treatment system. In addition to environmental problems caused by dairy effluent, the presence of milk solids in the effluent indicates the loss of a valuable product from the dairy industry. By recycling and reusing these types of effluents, these disadvantages are partially compensated. The dairy industry is one of the largest producers of effluents, the separator effluent of which is called dairy sludge and contains high amounts of organic compounds such as carbohydrates, proteins, that can be used as a source of carbon and nitrogen by microorganisms (Porwal et al., 2015). Dairy sludge has a volume between 0.5% and 1% of milk volume (about 1,000 tons per year), of which 14%–16% is dry matter. Dairy sludge contains 6%–8% nitrogen, 0.25%‐–35% fat, 4.7% lactose and 1.5%–3% non‐milk. Therefore, this compound can be used as a cheap and useful culture medium for the growth of microorganisms and the production of a variety of products (Porwal et al., 2015).

The aim of this study was to use different percentages of dairy sludge base culture medium as a carbon source and soybean meal treatments as a source of glutamic acid and mineral salt of ammonium sulfate to optimize GABA production in this culture medium by *L. brevis* PML1. For this purpose, the safety of this strain was assessed for future and nutritional use. Then, by determining the effect of dairy sludge, soybean meal and ammonium sulfate percentage in three levels and 95% confidence level, optimization was performed. Experimental of CCD and data analysis were performed by modeling of second degree of RSM of Design Expert software. The physicochemical and microbial properties (antimicrobial, antioxidant and toxicity against cancer cell) of the fermented extract were also investigated.

## 2 MATERIALS AND METHODS

### 2.1 Materials

The chemical materials used in this study include MRS, Mueller Hinton Agar (MHA), Violet Red Bile Agar (VRB) and compounds such as iron chloride, BHA, tetrazolium, sodium acetate, tetrahydrofuran, KOH, TCA, and ammonium sulfate from Merck Germany and Dulbecco's modified Eagle medium (DMEM), Fetal Bovine serum (FBS), were provided from Gibco (UK).

### 2.2 Activation of *L*. *brevis* PML1

In this study, in order to investigate the ability to produce GABA, the native *L*. *brevis* PML1 strain isolated from Tarkhineh, which had been isolated in previous research, was used (Vasiee et al., 2018). In order to activate, the lyophilized bacteria was transferred to the MRS Broth culture medium and heated at 18°C for 18 hr. Activated microorganisms were transferred to the MRS Agar culture medium and incubated at 24°C for 24 hr. This culture medium was used as a storage medium for subsequent tests. The purity of the desired strain was investigated by staining (Falah et al., 2019).

### 2.3 Fermentation

#### 2.3.1 Preparation of medium culture

The dairy sludge used in this study was prepared from Pegah Khorasan Razavi Dairy plant, Mashhad, Iran. The initial pH of the dairy sludge samples was set to about 6, and was heated for 5 min at 90°C to precipitate insoluble proteins and reduce microbial load. Then, in order to harmonize the composition of the culture medium and create favorable conditions, dairy sludge with semi‐industrial spray nozzle dryer, two flow nozzle, counter‐current (Soroush Sanat‐Iran), inlet air temperature of 180°C and inlet flow rate of 44.5 ml/min, was powdered (Liu et al., 2016). Soybean meal was also prepared from the Department of Animal Sciences, Faculty of Agriculture, Ferdowsi University of Mashhad, and after crushing by mill, it was passed through a 2 mm sieve to remove impurities and insoluble fibers. Initially, 3 levels of separator sludge powder and 3 levels of soybean meal powder along with 3 levels of ammonium sulfate were selected as mineral salts of the culture medium for GABA production and were prepared in 50 ml volume according to the statistical design. Magnet and hot plate were used to improve the solubility of the samples. All samples were passed through a vacuum filter with grade 541 filter paper and the filtered solution was autoclaved and sterilized for 15 min at 12°C in semi aerobic condition (Mukherjee et al., 2016).

#### 2.3.2 Fermentation process

One hundred μl (10^8^ CFU/ml) of activated *L*. *brevis* PML1, was transferred to Falcon containing 5 ml of culture medium prepared according to the statistical design at pH 6 and placed in a 32°C incubator for 120 hr (Dikshit & Tallapragada, 2015).

### 2.4 Effect of pH

Sampling and pH were measured every 24 hr evaluate the pH change process by pH meter‐ HANNA Portugal (Limón et al., 2015).

### 2.5 Evaluation of GABA production

#### 2.5.1 Thin layer chromatography/Spectrophotometry

After 120 hr of fermentation, the supernatant was centrifuged at 4°C and 11,200 *g* for 10 min. Then, the supernatant was filtered by a 0.22 µm filter. To evaluate the production of GABA, an active silica gel plate with dimensions of 20 × 20 cm was used. According to Kook and Cho (2013) method, the plate was first drawn horizontally in one direction and at a distance of 2 cm from the bottom with a pencil points were marked by one centimeter distance. It was then stamped by a 2 µl capillary tube on the plate. In this test, pure GABA solutions and bacterial culture medium were used separately as control (Kook & Cho, 2013). A solution consisting of ninhydrin with a combination of butanol, acetic acid and distilled water in a ratio of 2:3:5 was used as the mobile phase. After the band was identified, for 80 min, it was placed in the 70°C oven. Then the bands in front of the GABA were cut and dissolved in 75% ethanol solution and 0.6% copper sulfate (2:38 ratio) separately. The mixture was then placed in a 50 rpm incubator for 40 min at a temperature of 40°C. The absorption of each sample was then read at 512 nm. Due to the adsorbents read by the solution containing pure GABA and drawing the standard curve and equation, a small amount of GABA was obtained in the fermented extract of the samples (Jang et al., 2015; Kook & Cho, 2013).

#### 2.5.2 High‐performance liquid chromatography

After finding the optimal treatment with the TLC method, the GABA level in the optimal treatment was measured by the HPLC based on the method of Liu et al. (2016). The sample was centrifuged for 15 min at 1,008 *g* and the supernatant passed through a 0.45 µm filter. The filtered sample was added to 0.2 mM sodium bicarbonate at pH 9.8 in a 9:1 ratio. Then a dansylated dansyl chloride of 8 g/L was added to the sample and placed for 1 hr in 30°C (no light). GABA standards were prepared and dansylated at 250 and 450 mg/L concentrations. Analysis was performed using HPLC with column C18 (250 × 4.6 mm). The mobile phase consists of two phases A (sodium acetate 50 mM, methanol, tetrahydrofuran in a ratio of 5:75:420) and phase B (methanol) mixed with linear gradient from 1% to 100%. The mobile phase was injected into the column at a flow rate of 1 ml/min. A UV detector with a wavelength of 254 nm at 40°C was used to identify the output material from the column (Liu et al., 2016).

### 2.6 Safety evaluation

#### 2.6.1 Hemolytic activity and DNase production

Hemolytic activity of the extract was incubated for 48 hr in a blood agar culture medium (7% by weight of sheep’s blood weight) at 37°C. If the green color appear that is alpha hemolysis, or if there is no change, it is gamma hemolysis, which is considered to be without hemolytic properties. If a clear zone around the colony was observed, it is classified as beta hemolysis (Casarotti et al., 2017). Also, in order to investigate the production of DNase enzyme, strain was cultured in DNase culture medium. After incubating the plates at 37°C for 48 hr, clear pinkish colonies indicate the production of DNase (Hossain & Ahmed, 2017).

#### 2.6.2 Antibiotic susceptibility

In this study, the antibiotics of vancomycin (µg/ml) 30, phosphomycin (µg/ml) 20, kanamycin (µg/ml) 30, gentamicin (µg/ml) 10, neomycin (µg/ml) 30, cefixime (µg/ml) 5, ciprofloxacin (µg/ml) 5, ampicillin (µg/ml) 10 and erythromycin (µg/ml) 15 were used. This test was performed based on a Georgieva et al. (2015) method (Georgieva et al., 2015). First fermented extract was cultured in plates containing MRS Agar medium and then the antibiotic discs with specified concentration were inserted at a distance of 2–4 cm. Then we fixed them in 15 min at room temperature and after that incubated at 37°C for 24 hr. At the end of incubation the diameter of the clear zone around the disc was measured and according to the standard criteria, resistance and sensitivity to antibiotics were evaluated. The MRS Agar environment was used for positive control growth.

### 2.7 Cholesterol removal rate

The extract was cultured in MRS medium with 0.3% oxalate bile salt and 100 μl/ml cholesterol at 37°C for 28 hr. Bacterial fermentation extract was centrifuged at 4,032 *g* for 8 min at 4°C. 0.5 ml of supernatant was added to 3 ml of 95% ethanol and 2 ml of 50% KOH and mixed for 10 min 60°C in a steam bath and heated. Then 5 ml of hexane and 3 ml of distilled water were added. It was kept for 15 min to double phase of the mixture. Evaporation was performed using 2 ml of hexane at 60°C. 2 ml of o‐phthalaldehyde reagent was added and after 10 min, and 1 ml of concentrated sulfuric acid was added too and mixed. After 10 min, the absorption rate was read at 550 nm. The standard curve was used to determine cholesterol concentration (Choi et al., 2015).

Cholesterolremovalrate(%)=[(A1‐A2)/A1]∗100

*A*1 is the cholesterol in uninoculated culture, *A*2 is the cholesterol in supernatant.

### 2.8 Antimicrobial potential against food spoilage pathogenic bacteria

To prepare the bacterial extract for antimicrobial properties, the optimal treatment extract was first filtered to remove bacterial cells centrifuged for 20 min and 2,800 *g* at 5°C and supernatant passed through 0.22 μm filter paper and by using sodium hydroxide reached to pH 7, and finally lyophilized with a BETA LCS plus 2–8 freeze‐dried under freezing temperature of −45°C and heated to 32°C under 0.38 mbar vacuum for 40 hr. The dried sample was re‐dissolved with 4 ml of sterile distilled water and its antimicrobial properties were investigated by minimum inhibitory concentration by method of Behbahani et al. (2019) and diffusion in agar by method of Cizeikiene et al. (2013). The pathogenic strains used in this test included *E. coli* ATCC 25922, *Staphylococcus aureus* ATCC 25923, *Pseudomonas aeruginosa* PTCC 1707, *Salmonella typhimurium* PTCC1609, *and Listeria* *innocua* ATCC 33090.

### 2.9 Antioxidant properties

Optimal sample Antioxidant activity of the optimal sample was investigated using two methods of reducing power and DPPH radical scavenging activity.

#### 2.9.1 Reducing power

In order to evaluate the regenerative strength of the optimal sample, 1 ml of the sample was mixed with 1 ml of distilled water and 1 ml of potassium ferrocyanide (1%), and the resulting solution was added. After heating the solution for 20 min in a water bath at 50°C, 2.5 ml of trichloroacetic acid was added to it and was stir again. The solution was centrifuged at 750 rpm for 5 min. Then 2 ml of supernatant was mixed with 2 ml of distilled water and 1 ml of ferric chloride (FeCl3) 0.1% and after all mixing and storage for 10 min at room temperature, the adsorption of the solution was recorded at 700 nm. The results were reported as absorption unit (AU) (Nooshkam et al., 2019).

#### 2.9.2 DPPH‐Radical Scavenging (DPPH‐RS) activity

In order to evaluate the optimal free radical inhibitory activity, 2 ml of the sample was mixed with 1 ml of 0.2 mmol DPPH radical solution in 95% ethanol. After keeping the sample for 30 min at room temperature in a dark place, its absorption was recorded at 517 nm (Cai et al., 2014).

DPPHradicalinhibitoryactivity=[(1‐A1)/A2]∗100.

A1 is the adsorption control, A2 is the adsorption sample.

Ethanol BHT solution with a concentration of 15 mg/ml was used as the standard antioxidant and for comparison of the antioxidant activity of the samples.

#### 2.9.3 Cytotoxic properties

The cytotoxic effect of fermented extract was measured against Caco‐2 cancer cell line by MTT assay. The cells (Bu Ali Research Institute of Mashhad, Iran) were cultured in DMEM (Dulbecco’s Modified Eagle Medium) high glucose medium supplemented with fetal bovine serum (10% v/v) and penicillin/streptomycin, and incubated at 37°C under constant humidity 95% and 5.0% CO2 pressure. Caco‐2 cells were seeded in 96‐well flat‐bottom plates (approximately 100,000 per well) until 50%–60% confluence was achieved. The medium was then replaced with a complete culture medium containing DMEM and fetal bovine serum (200 μl) and various concentrations of fermented extract (to 200 mg/ml) were added to each well. The blank medium was regarded as control medium. The cell proliferation was quantified by MTT 3‐(4,5‐dimethylthiazol‐2‐yl)‐2,5‐diphenyltetrazolium bromide assay after 24 hr incubation time as follows. The MTT solution (30 μl; 5.0 mg/ml) was added to each well and the plates were incubated for 3.0 hr in a CO_2_‐equiped incubator. After removing the medium gently and adding DMSO (200 μl) into the wells, an ELISA/microplate reader at 570 nm reference filter was used to record the absorbance of the mixture. The fermented extract concentration (mg/ml) that was able to inhibit the cell growth by 50%, was calculated and defined as IC50. The cell viability curves were plotted with regard to the control cells (Behbahani et al., 2019).

#### 2.9.4 Statistical design

The statistical design in this study was the response level method using the Central Composite Design to optimize the variables affecting the dependent variable (GABA production). The mean GABA produced through twice the replication of each experiment was considered as the dependent or response variables. Data analysis and preparing the figures were performed with the second‐degree modeling of Design Expert version 8.0.0 software.

## 3 RESULTS AND DISCUSSION

Previous research has shown the probiotic properties and viability of strain in the simulated gastric and intestinal conditions as well as bile salts. Also, the hydrophobicity potential of this strain was 18.33%. The ability of *L. brevis* PML1to adhere to the intestinal cell line, Caco‐2 was determined 8.7% which was also confirmed by scanning electron microscopy. The results showed that co‐aggregation of the strain was 62%, and it was able to compete (43%), inhibit (39.5%) and displace (21%) the adhesion of *E. coli* to Caco‐2 cells and it was investigated in master thesis of Falah 2019.

### 3.1 Optimization of fermentation and GABA production

At the beginning of fermentation, pH decreases due to glucose uptake and the production of organic acid, and after a while due to H + consumption as a result of GABA production, the pH increases logarithmically. In acidic conditions, pH between 4 and 5, due to increased GAD activity, GABA production increases so that pH reduction can be considered as a criterion for evaluating enzyme activity (Ko et al., 2013).

According to the results, dairy sludge is a good environment for the growth of bacteria, which can also be used in fermentation. Due to the physical condition of this compound as well as the combination of the culture medium compounds, the drying process was done by spraying dryer. Controlling the properties of the powder and creating favorable conditions for estimating different needs as well as product uniformity, spray drying was used (Liu et al., 2016).

**FIGURE 1:**
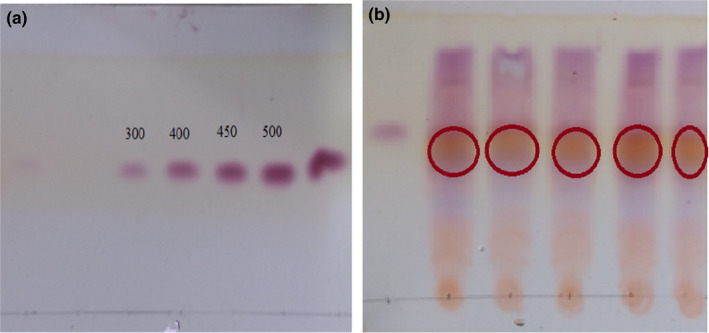
Thin layer chromatography, Standard concentrations (a). Samples (b)

Paper chromatography, or thin‐layer chromatography (TLC) is suitable for simultaneous analysis of a large number of samples (Figure 1). In this method, ninhydrin is used as the most important compound to identify GABA. Ninhydrin releases amines as well as carboxylic and amino acids bind to ninhydrin through the free electron pair of nitrogen amines. Then, by removing a CO_2_ molecule and binding the carboxyl group to decarboxylation, the amino acid is converted to an aldehyde compound and the ninhydrin itself to hydrindantin, forming a pink Roman complex (Kook & Cho, 2013; Yunes et al., 2016).

In order to determine the changes of GABA production, the effect of each of the independent variables, initially is needed to determine the appropriate modeling to fit the test data. Statistically, a model is suitable that significantly fit the data and the *R* ^2^ have the highest value. Due to the significance of the fitness test for production as well as the modified values of *R* ^2^, the second‐order multitasking model was selected to investigate the changes of the measured responses in this study. To determine the response level model, the result of linear, second‐order answers and the interaction of independent variables were used. The following equations show the experimental relationship between GABA production efficiency and independent variables with real numbers:

GABA = 268.2 + 36.65A + 17.55B + 18.45C – 0.6875AB – 8.44AC + 2.44BC – 19.53A² – 23.53B² + 6.97 C²

In which *A*, *B*, and *C*, are the linear effects, *A* ^2^, *B* ^2^, and *C* ^2^ are the effects of squares, and *AC*, *BC*, and *AB* are the interaction effects.

Because carbon has a direct effect on the properties and yield of fermentation, and it is the most important compound in the culture medium used to produce microbial metabolites. Lactose is the main carbohydrate in dairy sludge, which is the main nutrient for growth, and is an alternative source of carbon for growth and metabolites production. It seems that the reason for the effect of the carbon source on the GABA production is the high demand of the strain for the carbon source. (Mead et al., 2013). Singh et al. (2013) examined dairy sludge as an alternative crop medium for *Rhizobium* growth. The maximum growth of all strains was observed in sludge concentration of 65%. (Singh et al., 2013). Due to the high percentage of glutamic acid in soybean meal (50% protein and about 18% glutamic acid), this compound was used in the culture medium. Glutamic acid is the substrate for the decarboxylation reaction of GABA production, and it is obvious that with increase of its percentage, the amount of GABA production will increase. Soybean meal, in addition to protein, contains many fibrous compounds that may interfere with the fermentation process and reduce production efficiency. As a result, GABA production has declined at concentrations above 6% of soybean meal. According to a study by Chi and Cho (2016), soy‐based products, due to their short chains and bioactive peptides available for microorganisms, could be used in fermentation cultures to produce various amino acids (Chi & Cho, 2016).

Ammonium sulfate is a useful mineral salt for use in fermentation cultures. Due to the increase in hydrophobic bonds in the GAD enzyme, this compound increases the activity of the enzyme and increases the yield (Peyton et al., 2016). It is observed that with increasing percentages of dairy sludge, GABA production increased with a gentle slope. Followed by an increase in the percentage of dairy sludge to about 15%, the process of increasing GABA production was increasing due to carbon source which increases the efficiency of fermentation. Singh et al. (2013) examined dairy sludge as an alternative culture medium. The maximum growth of all strains was observed in 65% dairy sludge concentration.

**FIGURE 2:**
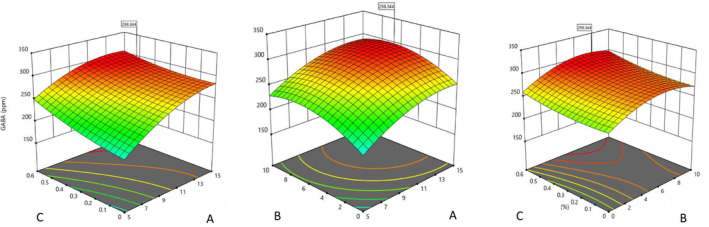
Three‐dimensional curve of treatments interaction effects on GABA production, (a) Dairy sludge (%), (b) soybean meal (%) (c) ammonium sulfate (%)

With increasing concentrations of ammonium sulfate, GABA production increased linearly (Figure 2). Maximum GABA production was observed at the maximum concentration of ammonium sulfate (0.6%). Ammonium sulfate causes catalytic activity for the enzyme by increasing hydrophobic bonding and minor changes in the structure of the active site (Ko et al., 2013; Kook & Cho, 2013). The results show that the GABA production phase improves with increasing carbon and nitrogen sources. Given that these two energy sources are the main component of GABA production, further production seems to be related to the increase in carbon and nitrogen sources to the consumption of these resources in the next phase of growth (stagnation phase).

The highest production of GABA by *L*. *brevis* took place in a medium containing 14.77% of milk sludge with 6.27% of soybean meal and 0.49% of ammonium sulfate (temperature 32°C for 120 hr) which produced 300 mg/L GABA.

**FIGURE 3:**
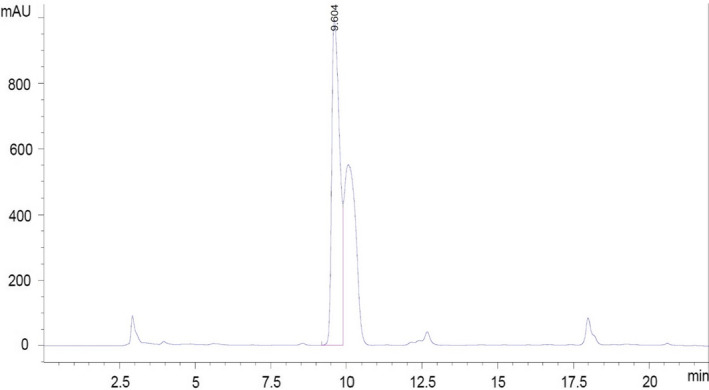
Monitoring the GABA production by HPLC chromatograms of fermented extract

In order to validate the model, the optimal point verification experiments were performed by re‐measuring the production efficiency of optimum condition and comparing it by HPLC. The maximum output was about 289 ppm (Figure 3). According to the GABA internal standard injection, the average retention time for GABA was 9 min and 60 s. There are a number of uncertain peaks in chromatograms that are related to other compounds used in the derivative stage of the sample. After determining the GABA peak and using different concentrations of GABA, the standard curve and consequently the linear regression equation *y* = 446.04*x* – 442.08 with determination coefficient of *R* ^2^ > .9997 were performed.

Process optimization is one of the most important activities in today’s competitive industry. In order to mass‐produce the factors affecting the production of amino acids, it is also necessary to optimize the industry for its commercial production. On the other hand, the high cost of research requires the use of methods that make it possible to determine the variables that affect a process with the least number of experiments, which is done using classical methods and designing statistical models. In this study, it was found that the RSM can be used to find the optimal conditions of the system (Hayat et al., 2014).

### 3.2 Safety assessment

**FIGURE 4:**
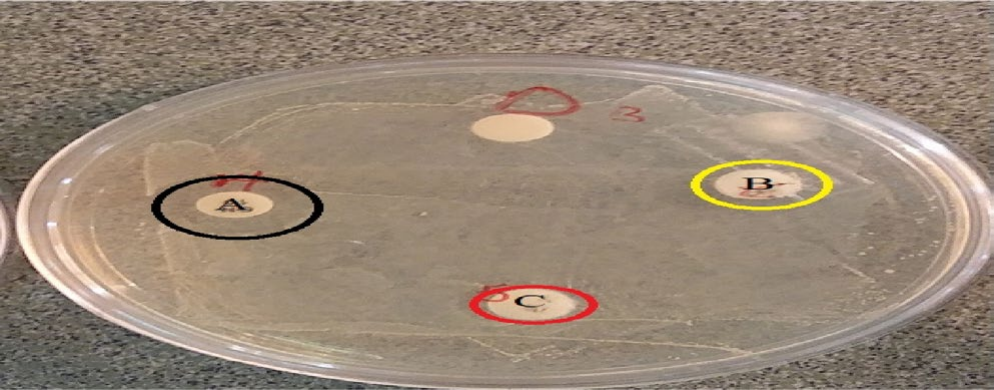
Effect of erythromycin (a) ampicillin (b), and gentamicin (c) on *Lb. brevis* PML1

In order to evaluate the safety of fermented extract, hemolytic activity, DNase production and sensitivity to various antibiotics were performed. Hemolytic or DNase activity was not shown which prove the safety of the strain. Another feature that is considered for the safety of extract is their resistance to antibiotics. The mechanism of action of antibiotics on the destruction of microorganisms varies. Some antibiotics prevent the formation of mRNA, and some, such as ampicillin, vancomycin and penicillin, destroy the bacterial cell wall. Beta‐lactam antibiotics such as penicillin and cephalosporins affect cell permeability and lead to wall degradation. The resistance and sensitivity of the strain to various antibiotics are shown in Table 1 and Figure 4. The fermented extract is resistant to gentamicin and vancomycin and can be tested in culture‐based tests of these antibiotics in the *L. brevis* culture‐specific culture medium. In the case of probiotics, resistance to vancomycin is important because it has a specific function against acute infections caused by pathogens resistant to combination drugs (Gueimonde et al., 2013; Vasiee et al., 2018). Abdulla et al. (2017) showed that *L. brevis* isolated from fermented vegetables resisted erythromycin and tetracycline (Abdulla et al., 2017). Cammarota et al. (2014) examined the resistance of *L. brevis* to kanamycin and clindamycin and showed that *lactobacilli* extract are generally resistant to aminoglycoside compounds due to enzymatic degradation and mutation and ribosomal changes that cause changes. It is known that resistance to antibiotic for LABs can have negative consequences. This resistance can be transmitted to the pathogens which presented in the intestine environment and make them resistant to antibiotic therapy. Although, in some strains resistance to antibiotics can be considered intrinsic and thus non‐transmissible. This feature needs further investigation in the case of the studied strain.

**TABLE 1:** Antibiotic susceptibility profile of potential *L. brevis* PML1

S. No	Antibiotics	Concentration (μg)	Fermented extract
1	Gentamicin	10	R
2	Vancomycin	30	R
3	Ciprofloxacin	5	I
4	Ampicillin	10	I
5	Erythromycin	15	S
6	Cefixime	5	S
7	Neomycin	30	S
8	Kanamycin	30	S
9	Fosfomycin	20	S

I, Intermediate; R, resistance; S, susceptible.

### 3.3 Cholesterol removal

High cholesterol is one of the leading causes of cardiovascular disease and the most common cause of death in the world. Although drug therapy can effectively lower cholesterol levels, long‐term use of the drug can lead to dangerous side effects. So one of the most effective ways today is probiotic therapy. In the present study, the rate of cholesterol reduction of *L. brevis* PML1 strain was 44% in an environment without bile salts, which is in accordance with the research of Li et al. (2013). Cholesterol absorption can be attributed to a variety of factors, including the production of coenzyme A, 3‐hydroxy‐3‐methylglutaryl, and the hydrolysis activity of bile salts. Also, in the study by Shehata et al. (2016) the absorption of cholesterol by *L*. *rhamnosus* was reported to be 33%, and the main reason was the effect of the production of fatty acids, especially propionate, on cholesterol absorption [25] and [27].

### 3.4 Antimicrobial properties of fermented extract in optimum conditions

*S. typhi*, with a minimum inhibitory concentration of 350 µg/ml, was the most susceptible strain and *L*. *innocua*, was the most resistant bacteria without any inhibitory concentration. In the well diffusion method, *S*. *typhi* and *E. coli* at a concentration of 500 mg/ml showed significant halo (12 and 14 mm) In addition, according to reports of Georgieva et al. (2015) antimicrobial properties of wheat fermented extract of *Lactobacillus* *plantarum* on *Streptococcus* *mutans* was reported which is due to the antimicrobial effect of lipoproteins produced during fermentation and prevention of biofilm formation (Georgieva et al., 2015). According to Nooshkam et al. (2019), cationic antimicrobial peptides have the potential to bind to lipopolysaccharides with negative charge (in gram‐negative bacteria) and teichoic and lipoteichoic acids (in gram‐positive bacteria) [32].

### 3.5 Antioxidant properties

Investigating the reducing power as an effective antioxidant method is to assess the ability of an antioxidant compound to donate an electron. This method is based on the reduction of iron chloride–ferrocyanide complex in the form of ferrous iron by antioxidants. The concentration of ferrous iron produced by measuring the soluble of Prussian blue color at 700 nm. The optimal sample had significant reducing power, but showed less antioxidant activity compared to BHA which is also consistent with Behbahani et al. (2019). The reducing power of the optimal sample can be due to the large number of hydrogen ions produced during fermentation (Table 2). Liu et al. (2016) showed that decarboxylation and enzymatic hydrolysis lead to the breaking, opening, and subsequent emergence of amino acids with the ability to donate electrons. These amino acids can react with free radicals to form relatively more stable compounds and stop free‐radical chain reactions. Low molecular weight peptides have been reported to have a high ability to neutralize DPPH radicals. In addition, the presence of tyrosine amino acids in terminal C peptide has been shown to be necessary for the radical neutralizing effect of some peptides [35].

**TABLE 2:** Antioxidant properties of fermented extract

Antioxidant properties	BHA	Optimization
Reducing power	83/0 ± 39/1	01/0 ± 47/0
DPPH	16/2 ± 37/72	79/10 ± 67/59

Hossain and Ahmed (2017) in a study on the antioxidant properties of whey protein products and some sugars introduced hydroxyl groups as a reducing agent stated that these products play an important role in breaking down the radical chains by donating electrons (Hossain & Ahmed, 2017).

### 3.6 Cytotoxicity

**FIGURE 5:**
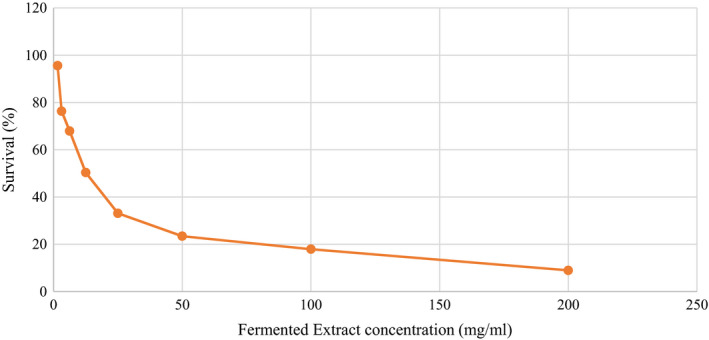
Cytotoxic effect of various concentrations of fermented extract on Caco‐2 cell line after 24 hr

The MTT method is used to assess the effects of cellular toxicity. Figure 5 shows the cellular toxicity of fermented extract on Caco‐2 cancer cell after 24 hr. The cytotoxicity effect was dependent on the fermented extract concentration; the higher concentration, the higher was cytotoxicity. It can be seen from Figure 5 that the highest percentage of cell viability was observed at 2.25 mg/ml fermented extract concentration, and a relatively low cell survivability was found when concentration was increased up to 200 mg/ml. The IC50 of the sample was 12.45 mg/ml. It can be confirmed by the MTT data that low concentrations of extract could stimulate cell proliferation substantially (*p* < .05). The toxicity of cancer cells was attributed to the production organic acids such as lactic and butyric, as well as the production of bioactive compounds such as GABA during fermentation, which leads to anti‐mutagenic properties.

## 4 CONCLUSION

Advances in understanding the link between nutrition and health to achieve an optimum health and reduce the risk of disease have led to the development of functional foods. In this study, the maximum production of GABA using *L*. *brevis* PML1 strain isolated from Tarkhineh in the culture medium containing 14.77% of milk sludge with 6.27% of soybean meal and 0.49% of ammonium sulfate (32°C and duration of 120 hr) which produced 300 mg/L GABA. The production of many amino acids by various chemical, enzymatic, methods is costly, so by using cheap raw materials in a biological way, the cost of production can be greatly reduced and its important that we use crude extract of GABA in fermented extract and, it can be used by GABA purification in future research. Findings from various studies show that the biological production of GABA by lactic acid bacteria, in addition to its benefits, is a bioactive, safe and environmentally friendly compound, which makes the production of enriched new products possible as well. Due to the biological nature of GABA produced in this study, it is possible to use this chemical to replace the chemical type in pharmaceuticals by purifying this amino acid. Also, by performing clinical tests on fermented extract containing GABA produced, it will be possible to produce functional food products. In addition, the rational use of industrial waste can help to reduce the environmental impact of the dairy industry.

## CONFLICT OF INTEREST

We wish to confirm that there are no known conflicts of interest associated with this publication.

## AUTHOR CONTRIBUTIONS

**Fereshteh Falah:** Data curation (equal); Formal analysis (equal); Investigation (equal); Methodology (equal); Resources (equal); Software (equal); Writing‐original draft (equal). **Alireza Vasiee:** Data curation (equal); Formal analysis (equal); Investigation (equal); Methodology (equal); Writing‐original draft (equal). **Behrooz Alizadeh Behbahani:** Investigation (equal); Methodology (equal); Resources (equal); Writing‐original draft (equal). **Farideh Tabatabaei Yazdi:** Funding acquisition (equal); Project administration (equal); Supervision (equal); Validation (equal); Writing‐review & editing (equal). **Seyed Ali Mortazavi:** Project administration (equal); Software (equal); Validation (equal); Writing‐review & editing (equal).

## Data Availability

All data generated or analyzed during this study are included in this published article and also all data that support the findings of this study are openly available.
